# New manioc allergens and successful oral immunotherapy in a Brazilian allergic patient

**DOI:** 10.1186/1939-4551-8-S1-A181

**Published:** 2015-04-08

**Authors:** Roberta Almeida Castro, Keity Souza Santos, Ariana Yang, Fabio Fernandes Morato Castro, Clóvis ES Galvão, Adriano Mari, Fatima Ferreira, Gabriele Gadermaier, Jorge Kalil

**Affiliations:** 1 University of São Paulo School of Medicine – FMUSP, Brazil; 2 University of São Paulo, Brazil; 3 Idi-Irccs, Italy; 4 University of Salzburg, Austria

## Background

Exotic tropical fruits and plants are highly consumed in Brazil and due to globalization these products are being exported worldwide. We have recently described the first manioc allergen Man e 5 that cross-reacts with Hev b 5 from latex. There are more than 70 products made up manioc starch such as drugs, soaps and fabrics and manioc allergic patients present from mild to severe reactions.

## Methods

Five patients with manioc allergy confirmed by skin prick test (SPT) and oral challenge were selected. These patients were evaluated through SPT and Immunocap to latex, ELISA and ISAC inhibition with manioc extract and rMan e 5. Among these patients we selected a Brazilian woman that lives in North region where manioc is consumed in a daily diet for an oral immunotherapy (OIT) protocol. The patient is a hairdresser, 48 years old, IgE mediated latex allergy. Six years ago she started to have episodes of anaphylaxis to manioc, but more recently she began to present allergic manifestations after inhalatory exposition to manioc starch. Patient was then submitted to OIT with manioc starch solubilized in 10mg/mL. Immunotherapy was divided in two parts: induction and maintenance. In the first phase dose was weekly increased starting with 0.1mL of a 10mg/mL until reaching an amount of 10g of manioc starch. At this point the patient was submitted to oral provocation with 100g of manioc. For maintenance it was indicated daily ingestion of foods prepared with manioc. Clinical evaluation, skin tests and IgE levels after 1 and 6 months were performed.

## Results

All five patients had anaphylaxis to manioc with IgE sensitization to manioc and latex. Inhibition assays showed that rMan e 5 is not the only manioc allergen and that there is a molecule in manioc cross-reacting with Hev b 6. After OIT patient presented a decrease of the wheal in SPT (10 to 4mm) and also in IgE levels to latex. Oral provocation was negative. Reactions during treatment were mild (abdominal pain and oral itching) and solved spontaneously or using antihistaminic. After two years under maintenance patient keeps asymptomatic eating manioc without restrictions.

## Conclusions

There are at least two manioc allergens cross-reacting with latex. OIT was proved to be safe and efficient in this case allowing free ingestion of manioc leading us to conduct the protocol with other patients. Levels of latex IgE also decreased after treatment. Knowledge of single allergens is important for diagnosis and also to monitor therapeutic success.

